# Coherent movement of error-prone individuals through mechanical coupling

**DOI:** 10.1038/s41467-023-39660-6

**Published:** 2023-07-18

**Authors:** Federico Pratissoli, Andreagiovanni Reina, Yuri Kaszubowski Lopes, Carlo Pinciroli, Genki Miyauchi, Lorenzo Sabattini, Roderich Groß

**Affiliations:** 1 Department of Sciences and Methods for Engineering, University of Modena and Reggio Emilia, Reggio Emilia, Italy; 2 Department of Automatic Control and Systems Engineering, The University of Sheffield, Sheffield, UK; 3 IRIDIA, Université Libre de Bruxelles, Brussels, Belgium; 4 Department of Computer Science, Santa Catarina State University, Joinville, Brazil; 5 Department of Robotics Engineering, Worcester Polytechnic Institute, Worcester, MA USA

**Keywords:** Computer science, Electrical and electronic engineering

## Abstract

We investigate how reliable movement can emerge in aggregates of highly error-prone individuals. The individuals—robotic modules—move stochastically using vibration motors. By coupling them via elastic links, soft-bodied aggregates can be created. We present distributed algorithms that enable the aggregates to move and deform reliably. The concept and algorithms are validated through formal analysis of the elastic couplings and experiments with aggregates comprising up to 49 physical modules—among the biggest soft-bodied aggregates to date made of autonomous modules. The experiments show that aggregates with elastic couplings can shrink and stretch their bodies, move with a precision that increases with the number of modules, and outperform aggregates with no, or rigid, couplings. Our findings demonstrate that mechanical couplings can play a vital role in reaching coherent motion among individuals with exceedingly limited and error-prone abilities, and may pave the way for low-power, stretchable robots for high-resolution monitoring and manipulation.

## Abstract (web summary)

In biology, individuals are known to achieve higher navigation accuracy when moving in a group compared to single animals. The authors show that simple self-propelled robotic modules that are incapable of accurate motion as individuals can achieve accurate group navigation once coupled via deformable elastic links.

## Introduction

A variety of animals, including humans, can achieve higher navigation accuracy by moving as a group than they would do as single individuals^1–6^. This phenomenon has been described as the many-wrongs principle, where individual errors are filtered out by mechanisms of information pooling. Information transfer within groups of moving animals has been conveniently modelled using artificial forces, where individuals are assumed to be capable of computing force vectors from the relative position of nearby individuals and determining their own movements as the summation of these vectors^7,8^. As a result of these movements, the groups may assume spatial formations such as regular lattices or triangulations^9–11^. These formations of individuals can behave like active elastic sheets^9,10^, and navigate confined spaces by translating, rotating, and shrinking/growing in size^11^.

Unlike most previous works on the many-wrongs principle, our work focuses on groups of individuals that are mechanically coupled via deformable links. The individuals are minimalist robotic modules, hence, the aggregates they form could be considered soft-bodied modular robots. We are specifically interested in modules that are ultra-low power and of limited size, and hence may be unable to navigate their environment effectively on their own. We hypothesise that a formation of such modules, once coupled via a deformable substrate, could coherently move, shrink, or stretch in size, by leveraging the underlying material properties to coordinate their motion. As coherent motion would no longer rely exclusively on the individuals’ ability to gather and pool information, and move accordingly (the approach taken by the vast literature on coherent motion^11–17^), the proposed system would be less reliant on (energy-intense) technologies for perception, computation, and actuation. This approach, which is fundamentally routed in embodied intelligence^18–21^, could have important implications for the design of distributed aggregates performing morphological computation at scale^22^.

Although coherent movement has been reported for aggregates of rigidly linked modules^23–29^, we are specifically interested in aggregates of modules connected via deformable links^30–32^, and the potential advantages they may offer in future applications. In the medium term, such aggregates could perform operations on objects of complex geometry and doing so with high spatial resolution. For example, they could move into tubular structures that are inaccessible to humans, scan the underlying surfaces for leakages and apply sealants^33,34^. In the long term, they could contribute to novel forms of stretchable, electronics technologies^35^, enabling flexible and morphable devices to deploy themselves within the human body^36^. For example, following delivery through a catheter, they could autonomously attach to, and wrap around, a damaged section of an organ, providing high-spatial resolution monitoring or treatment.

The system we propose is at the interface of multiple fields: (i) soft robotics^37^, due to the use of deformable materials to link the units, which enables the aggregates to stretch and shrink; (ii) modular robotics^38,39^, due to the modular and reconfigurable nature of the resulting aggregate; and (iii) swarm robotics^40,41^, due to the individual autonomy of the units, and the decentralised, leader-less algorithms they execute. Soft robotics has been a very active research field in the last decade^37,42–44^. It studies robotic systems made of deformable materials. The systems’ mechanical structures are inherently compliant. Therefore, soft-bodied robots promise a high degree of versatility and robustness, and are generally safer to interact with than robots of conventional design^45^. When soft-bodied robots collide with the environment, the impact is absorbed by their compliant structures, thus reducing damage to themselves, as well as the environment^46,47^. Although some soft-bodied robots are of a modular design^48^, the underlying segments are usually linked irreversibly at the fabrication stage. A less explored alternative are soft-bodied robots that are modular and reconfigurable. Given a set of reconfigurable building blocks—the modules—a vast amount of robot structures can be realised in a cost effective manner, potentially even by the robots themselves^49^. The shape and size of these structures could be changed to cope with new and unknown situations, and solve problems that would otherwise be impossible for single robots^50^.

Most of the research on soft-bodied modular reconfigurable robots has been devoted to the definition of mechatronic structures that allow modules to self-assemble. In ref. ^51^, spherical modules made of an elastic membrane containing gas are presented. The modules assemble using electrostatic forces. Two prototypes, weighing 1.5 g, were built and succeeded in self-assembling. In ref. ^52^, a group of rigid modules reside within an elastic membrane. Each module has a tail light and a light sensor. The movement of individual modules is restricted to 1-D. Despite these restrictions, simulations demonstrate that tasks that require complex 2-D movement can be realised by the aggregate. A hardware prototype is discussed. In ref. ^53^, a system of cube-shaped modules is presented, which, using permanent magnets, can be manually configured into different shapes. The modules are pneumatically actuated, however, are incapable of autonomous movement. Their movements are centrally controlled, with pressurised air provided via an external pump. The authors use 24 of these modules to conduct experiments^54^. In ref. ^55^, cylindrical modules made of deformable printed circuit boards are presented. They feature electro-permanent magnets that allow them to assemble. Although having no moving parts, a module can change position by interacting with another module; while doing so, the latter has to retain its position, but may change in orientation.

**Fig. 1:**
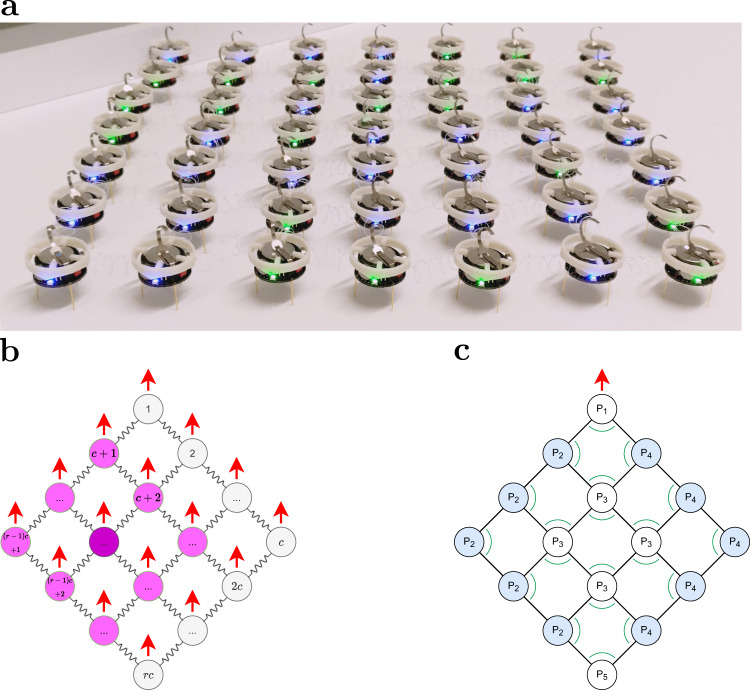
The Kilobot Soft Robot comprises a group of fully autonomous Kilobot modules that are arranged in a square lattice configuration and mechanically coupled via elastic links. **a** A 7 × 7 Kilobot Soft Robot composed of 49 modules (colours indicate status of battery) connected by 84 transparent springs (see Fig. S1 for a graphically enhanced photo highlighting the springs). **b** All modules are initially orientated in a common direction (indicated by red arrows). Each module can broadcast messages to the other modules within its Moore neighbourhood (e.g. discs indicated as magenta for the shaded one) and estimate the distance from neighbours. All modules have unique IDs (see labels). **c** Each module monitors for local deformations using a combination of distance and angle estimates. If residing on the left or right boundaries (*P*_2_ and *P*_4_; in blue), a module computes an angle perpendicular to the motion direction, whereas at the head (*P*_1_), interior (*P*_3_) or tail (*P*_5_), it computes angle(s) parallel to it.

This paper proposes the Kilobot Soft Robot, a novel soft-bodied modular reconfigurable robotic system. It extends the work in ref. ^30^, which originally outlined the idea and included preliminary results. Each module of the Kilobot Soft Robot is based on the openly available Kilobot platform^56,57^. The module features a custom-made circlet, allowing it to be mechanically coupled with other modules via elastic links (springs). The modules and springs assume a square lattice configuration (see Fig. 1a, b). The lattice dimensions, and hence, the default shape of the Kilobot Soft Robot, can be manually reconfigured to suit different tasks.

Unlike other soft-bodied robots, the Kilobot Soft Robot is actuated using vibration motors. The latter are inexpensive actuators commonly found in mobile phones. Each module moves using a pair of these motors, while being partially constrained in position and orientation by the springs. Each module can communicate with the modules in its Moore neighbourhood (see Fig. 1b). Depending on its position within the structure, it has three to eight neighbours to communicate with. It can determine the distance to any module from which it receives a message. On its own, however, it is unable to determine the bearings. By relying on simple, light-power and inexpensive hardware, the Kilobot Soft Robot, and adaptations thereof, could possibly be realised at a larger scale, with thousands of modules. Compared with the original, rigid Kilobot platform, which has been used to produce aggregates that move through progressive repositioning of boundary modules^58,59^, the Kilobot Soft Robot propels by the parallel displacement of all its modules—whether residing at the boundary or in the interior. Consequently, the speed by which Kilobot Soft Robots propel is not necessarily limited by the fraction of modules residing in its interior.

To test the properties of the Kilobot Soft Robot in practice, we conduct a series of experiments with up to 49 physical modules. To the best of our knowledge, this is one of the first demonstrations of a fully autonomous soft-bodied modular reconfigurable robot of this size (in terms of number of modules). While a previous study developed a larger self-propelling (rigid-body) modular aggregate comprising 55 modules^60^, no systematic experiments were conducted to characterise its performance. Ours is the first systematic study to show a large modular robot (being rigid or soft) to effectively self-propel and navigate in space. We also include a formal analysis of the elastic coupling between the modules, and discuss how the findings inform the design of soft-bodied modular reconfigurable robots.

## Results

We present three distributed algorithms that enable (i) modules to self-localise with respect to their neighbours, and Kilobot Soft Robots to (ii) move and (iii) deform. The same set of algorithms are executed by each module of the robot. They are based on the following assumptions:

- The modules are initially arranged in a square lattice configuration (hexagonal configurations can be considered through parameter modification, see Fig. S2).
- Each module can locally broadcast small messages (i.e. a few bytes) that are received by other modules within its Moore neighbourhood.
- When receiving a message, a module has the means to obtain a noisy estimate of its distance from the emitter. For the purpose of this study, the module requires no further sensors.
- As communication (and sensing) is isotropic, the module is unable to determine its orientation (unless when moving relative to its neighbours^58,59^). However, all modules share a common orientation provided the lattice configuration has not deformed.
- All modules have locally unique identifiers (IDs), and are aware of all IDs within their local neighbourhood. They use them (i) when broadcasting messages (i.e. include the emitter ID) and (ii) to determine which neighbours they are expected to receive messages from (i.e. modules at the boundary of the configuration have fewer neighbours). For the sake of simplicity, we opted to assign globally unique IDs 1, 2, …, *cr* to the modules within an *r* × *c* lattice (see Fig. 1b). This enables modules to automatically deduce their neighbours’ IDs from their own ID, *r* and *c*. As an alternative, algorithms from the literature could be used to enable modules to discover the scale of the configuration and self-assign position-based unique IDs^58,61^.

As part of the localisation algorithm, each module continuously exchanges messages within its neighbourhood via local broadcast. Messages from modules other than its neighbours are discarded. Based on the signal strength of the messages received, a module estimates the distances to its neighbours. Distance estimates are locally shared to determine the relative bearing among neighbouring modules, similar to ref. ^58^. This allows any module to self-localise with respect to its neighbours. Unlike refs. ^58,59^, the module can do so while its neighbours are in motion. Due to the modules being mechanically coupled, the complexity of the localisation problem is greatly reduced.

As part of the motion control and deformation control algorithms, each module monitors for local deformations in the structure, and if they occur, plans its movements accordingly. If deformations are detected along the lateral axis, a module will perform a corrective move by turning left or right. If deformations are detected along the longitudinal axis, a module may choose to stop movement, enabling other modules to catch up. Deformations naturally arise as a result of inaccurate and imprecise sensors and actuators. However, they also arise where the modular robot is tasked to move along a curved trajectory, as the distance a given module has to travel depends on how far it is away from the centre of curvature (and hence on its column within the structure).

The modules use a combination of angle and distance estimates to probe for local deformations within the structure. To do so effectively, the module deduces its principle position within the lattice (e.g. at the boundary, it will have fewer neighbours to coordinate with). A module can be in any of five principle positions, denoted as *P*_1_, *P*_2_, …, *P*_5_ (see Fig. 1c). Modules shown in white reside in head (*P*_1_), interior (*P*_3_) and tail (*P*_5_) positions. They have a symmetric neighbourhood, and compare the distances to neighbours on their left and right to determine whether a movement is necessary to correct for a lateral deformation (i.e. a turn towards their left or right side). They also monitor the angles shown in Fig. 1c to determine if they have excessively advanced along the longitudinal axis and hence should stop, allowing neighbouring modules to catch up. Modules shown in blue reside in boundary positions, on either the left (*P*_2_) or right (*P*_4_) sides and have an asymmetric neighbourhood. To determine whether a movement is necessary to correct for a lateral deformation, they compare both the angle shown in Fig. 1c and the distance to their lateral neighbour against reference values. They also monitor the distance to the neighbour(s) ahead of them, to determine whether to stop. Under ideal conditions, all modules of the aggregate would move forward with identical speeds, and no agent would be required to stop. In practice, some agents advance faster than others, due to individual differences in actuation and sensory noise. Moreover, when the aggregate moves along a curved trajectory, some agents have to travel further than others, depending on their distance from the centre of curvature. Although the use of deformable links reduces the extent of associated deformations, the stop behaviour plays an important role, enabling agents which would otherwise fall behind to catch up.

The motion control algorithm assumes right angles as the reference, thereby realising a square lattice configuration. The deformation control algorithm is identical to the motion control algorithm, apart from using a time-varying input as the reference instead. Details are provided in the ‘Algorithms’ section of the ‘Methods’.

We conducted a series of studies to evaluate the performance of the Kilobot Soft Robot in different configurations, including its ability to move in the absence of external cues, to follow a reference trajectory, to change the shape of its body while following a reference trajectory, and its performance for different levels of link rigidity.

In all studies, the distributed algorithms are responsible for controlling the movement and shape of the robot using only local communication among its modules (see ‘Algorithms’ section under ‘Methods’). The first experiment requires the Kilobot Soft Robot to produce coherent forward movement with respect to its initial pose. The motion control algorithm lets all modules perform their default behaviour—to move forward—provided they do not detect significant deformations in their local neighbourhood. The movements of a module (forward, and corrective turns, where applicable) are all within its local coordinate system. Two other experiments require the Kilobot Soft Robot to follow a reference trajectory. For these experiments, we opted to provide the head module (*P*_1_) with binary feedback on the Kilobot Soft Robot’s position relative to the trajectory. Hence, the head module moves according to the received feedback. The feedback is not shared with the other modules, which are oblivious to the external situation, and move forward by default. However, as the head module follows a curved trajectory, local deformations occur, which in turn prompt nearby modules to adjust their poses. The effect hence propagates through the aggregate, enabling the latter to follow the curved trajectory while maintaining its formation. Although our experiments confirm that a single module is sufficient to bias the movement of a relatively large aggregate towards a desired direction, as already acknowledged and modelled in animal^7^ and robot collectives^12^, this module also represents a single point of failure. In fact, in one of the trials of the deformation experiment, the front module exhibited a fault, which prevented the system from completing its task. As an alternative, feedback about the reference trajectory could be provided to a set of modules and the modules’ membership for this set could dynamically evolve over time.

Movie S1 in the Supplementary Information contains a collection of clips summarising the experiments. Moreover, video recordings from the experimental trials are available at 10.15131/shef.data.22269319.

Details about the physical platform and experimental setup are provided in the ‘Hardware platform’ and ‘Experimental setup’ sections under ‘Methods’.

### Straight motion by robots of different sizes

**Fig. 2:**
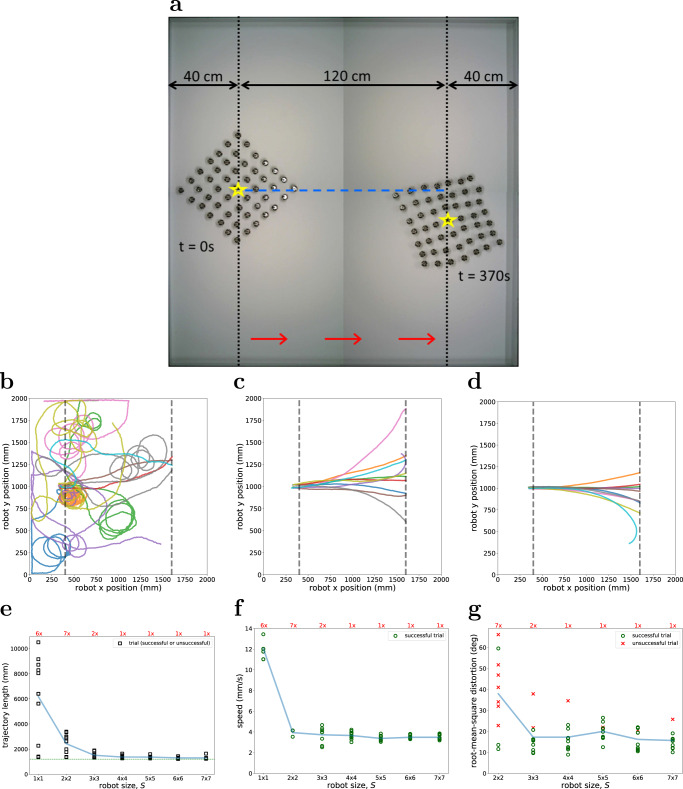
Physical experiment in which Kilobot Soft Robots of size *S* ∈ {1 × 1, 2 × 2, …, 7 × 7} are programmed to move straight for 120 cm in the absence of external feedback (10 trials per robot size). **a** Bird’s-eye view of the experimental area, generated by superimposing snapshots taken at the beginning and end of a typical experimental trial. The vertical dashed lines represent the start and finish lines. The robot’s position (i.e. its centre of mass, or CoM) is denoted by a yellow star. Trajectories of **b** 1 × 1 Kilobot Soft Robots (a single Kilobot with circlet), **c** 4 × 4 Kilobot Soft Robots, and **d** 7 × 7 Kilobot Soft Robots. Dashed lines represent the start and finish lines. **e** Length of Kilobot Soft Robot’s trajectory at the end of the trial (with the optimal length indicated as a green dotted line). **f** Linear speed of the robot along the trajectory it moves, which we computed using a finite resolution (10-s intervals). **g** Distortion of the robot’s body. In (**f**), only successful trials are reported. In (**e**–**g**), the text at the top reports the number of unsuccessful trials, whereas the blue lines indicate mean values.

This section reports an experiment to quantify the performance of Kilobot Soft Robots of different sizes when instructed to follow a straight reference trajectory. Figure 2a shows a bird’s-eye view of the experimental arena, which has dimensions of 200 cm × 200 cm.

Two snapshots showing the start and end of a typical experimental run are superimposed. The dotted lines indicate the start and finish lines, which are placed at a distance of 120 cm from each other. At the beginning of each trial, the Kilobot Soft Robot is positioned such that its Centre of Mass (CoM), computed as the average position of all modules composing the robot and indicated with a yellow star, is in the vertical centre of the start line. The Kilobot Soft Robot is oriented in parallel to the x-axis to directly face the finish line (on the right). It is tasked to move forward. It is not provided with any external feedback and does not perceive any cues related to the trajectory. In this sense, the robot is assessed in open-loop control. However, the modules estimate the relative positions among each other, and act accordingly.

We conduct experiments with robots of seven sizes, *S* ∈ {1 × 1, 2 × 2, …, 7 × 7}. For each size, 10 trials are performed, that is, 70 trials in total. Each trial is run for a fixed duration *T* = 800 s, which we chose by doubling the expected time for successful completion (in the absence of any faults) which was established through preliminary tests. If the robot (its CoM) reaches the finish line, the trial is considered successful and stopped. Otherwise, the trial is considered unsuccessful.

Only 40% and 30% of trials were successful for the 1 × 1 and 2 × 2 Kilobot Soft Robots, respectively, whereas 80% of trials were successful for the 3 × 3 Kilobot Robots. For Kilobot Soft Robots of larger size (4 × 4 to 7 × 7), 90% of trials were successful. Hence, a clear trend can be observed (see Table S1 in Supplementary Information).

Figure 2b–d show the trajectories taken by 1 × 1, 4 × 4 and 7 × 7 Kilobot Soft Robots, respectively, in all trials. It is apparent that the 1 × 1 robot (essentially a single Kilobot with open-loop control) is unable to move in a coherent direction. By contrast, the 7 × 7 robot deviates far less from the reference trajectory. Lacking feedback that is external to the robot, however, the robot will not stay on course indefinitely.

Figure 2e shows the length of the trajectory at the end of the trial as a function of the Kilobot Soft Robot’s size *S*. For each size, we report on the top the number of failed trials. The mean value (blue solid line) shows that the trajectory length decreases, converging to the optimum value (green dotted line), as the robot size *S* increases. In other words, the motion of the Kilobot Soft Robot becomes more accurate, the more modules it has.

The performance improvement in terms of accuracy comes at the expense of a reduced average linear speed, as shown in Fig. 2f. Although accuracy tends to improve with robot size *S*, the speed seems to settle to a constant value of about 0.35 cm/s (successful trials only). Comparing the robot’s speed with that of individual Kilobots (about 1.2 cm/s), we observe that the Kilobot Soft Robot moves at about 29% of the maximum speed of its constituent modules.

Finally, for each trial, we evaluated the extent to which the robots’ shapes were distorted with respect to the reference shape, a square lattice. In the reference shape, all angles formed between adjacent links to neighbouring modules and internal to the robot have the same value of 90°. The root-mean-square distortion is computed as the mean squared error from 90° for all angles. Figure 2g reports the average of the root-mean-square distortion over time. We observe relatively small values of distortion in successful trials (green circles) for robots of any size. Large distortions are observed in the unsuccessful trials (red crosses). The latter indicate that a high proportion of elastic links ended up not being in the correct configuration, which may cause the robot to cease motion.

### Following a reference trajectory

**Fig. 3:**
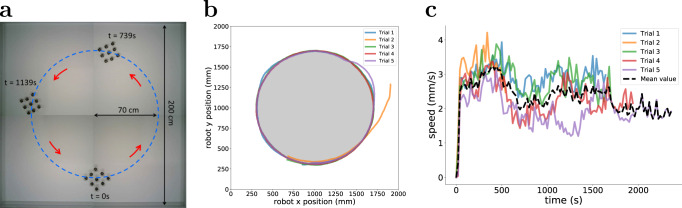
Physical experiment in which 3 × 3 Kilobot Soft Robots are programmed to follow a reference trajectory with closed-loop control. **a** Bird’s-eye view of the experimental area, generated by superimposing snapshots taken from a typical experimental trial. The reference trajectory (dashed circle) is a circle of radius 70 cm. **b** Motion trajectories of the robot (its CoM) in five trials. In four trials, the robot successfully completed the revolution. In one trial, it failed (Trial 2), hitting the right boundary. **c** Linear speed of the robot (its CoM).

This section reports an experiment that quantifies the performance of Kilobot Soft Robots when instructed to follow a predefined reference trajectory. The reference trajectory is a circle of radius 70 cm placed in the centre of the arena as depicted in Fig. 3a. A 3 × 3 Kilobot Soft Robot is initially placed on the reference trajectory, facing in the counterclockwise direction.

To enable the Kilobot Soft Robot to follow the reference trajectory, we opted to provide the module in the head position (*P*_1_) with feedback from an external localisation system. At 2-s intervals, the module receives one bit of information, indicating whether the reference trajectory is to the left or to the right of the robot (its CoM) with respect to its direction of motion. It chooses accordingly to either turn left or turn right for a brief period of time before returning to move forward. All other modules are oblivious to the external information and only tasked with moving forward; however, because they also respond to local deformations, the Kilobot Soft Robot can coherently move along the reference trajectory.

Five trials are performed. Each one is run for a fixed duration of *T* = 3600 s. If the robot (its CoM) completes one full revolution on the trajectory, the trial is stopped and considered successful. Otherwise, the trial is considered as unsuccessful. Trials where the robot collides with the boundary are aborted, and deemed unsuccessful. Figure 3b shows the trajectories for each trial. One of the trials (Trial 2) was not successful, as the Kilobot Soft Robot collided with the arena boundary. In all other trials, the Kilobot Soft Robot followed the reference trajectory with reasonable accuracy. Figure 3c shows the linear speed of the robot (at its CoM) over the duration of the five trials. The black line represents the mean value. Although the speed values were calculated with finite resolution (10-s intervals), the results show that the robot moved roughly with a constant speed, and did not cease motion for long (if at all).

### Changing shape while following a reference trajectory

This experiment quantifies the performance of the Kilobot Soft Robot when instructed to deform its body while following a predefined trajectory. The robot is required to first advance in its default shape, then shrink and finally expand again, to restore its original shape. Each module receives two signals by the external localisation system, indicating when the corresponding shape changes are to be triggered. In addition, the frontal module receives, at 2-s intervals, one bit of information, indicating whether the reference trajectory is to the left or to the right of the robot (its CoM) with respect to its direction of motion.

**Fig. 4:**
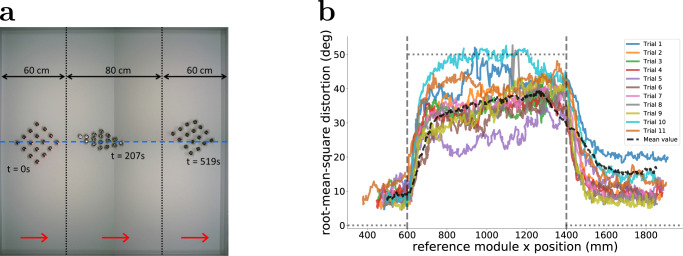
Physical experiments with 4 × 4 Kilobot Soft Robots that are programmed to move forward, first in a square lattice shape, then in a compressed shape, and finally relaxing to restore their original shape. **a** Bird’s-eye view of the experimental area, generated by superimposing snapshots taken from a typical experimental trial. The vertical dotted lines represent the positions where shrinking and expanding procedures are externally triggered, respectively. **b** Distortion, measured as the root-mean-square deviation of all internal angles from 90° for each of the 11 trials. The reference value is shown as a grey dotted line: the deviation is 0° for the first 60 cm (square lattice), 50° from 60 cm to 140 cm (stretched lattice), and again 0° thereafter. The black dashed line represents the mean value.

We use a 4 × 4 Kilobot Soft Robot, and conduct 11 trials. Figure 4a shows a bird’s-eye view of the experimental arena. The Kilobot Soft Robot is controlled to move along a straight (dashed) line, aligned with the x-axis. The initial and final positions for the robot (its CoM) are set at about 40 cm and 180 cm from the left, respectively. The vertical dotted lines, positioned at 60 cm from the left and 60 cm from the right, respectively, represent the positions where a change in the shape is to be triggered (as soon as the frontal module *P*_1_ reaches the corresponding line). Figure 4b shows, for each trial, the distortion with respect to the square lattice shape, measured during the experiment. The Kilobot Soft Robot successfully reached the final stage in all but one trial (Trial 3). The distortion values are computed as the mean squared difference from 90° for all the angles among the modules: Large values indicate that several angles were significantly different from 90°, implying that the body of the Kilobot Soft Robot is shrunk. The figure shows that the distortion was large when the body was expected to be shrunken, that is, when the position of the CoM was between the two vertical lines indicated in Fig. 4a. The shape change was relatively swift and was completed within a travelled distance of 10–20 cm. Once the robot reached the line that triggers the first change in shape (i.e. to shrink), the distortion values rapidly increased, almost to the reference value. When reaching the line that triggers the second change in shape (i.e. to restore the original configuration), the distortion rapidly reduced, though not entirely to the original value but to values comparable to experiments that did not require deformation (see Fig. 2g). Figure 4b also reveals considerable variability between trials. Due to significant variation in the ability of individual modules to self-propel (Fig. 2b), in relatively small aggregates (i.e. 16 modules) the variation between repetitions can be high. Nevertheless, the results show that in all but one of the trials the robot succeeded in shrinking and expanding its shape.

### Robots with different link rigidity

This section examines the role of the mechanical coupling on the performance of Kilobot Soft Robots.

To establish the utility of mechanical coupling, we perform a control experiment with a 2 × 2 Kilobot Soft Robot that has its (four) elastic links removed. Ten trials were conducted. In all trials, the group split. Nevertheless, in one of the ten trials, their CoM reached the finish line. The trajectories of the CoM are included in the Supplementary Information (see Fig. S3).

To establish the utility of elastic mechanical coupling, we perform two further control experiments where Kilobot Soft Robots have their elastic links replaced with rigid links of equivalent length and weight (see Fig. S4a). In the first experiment, we ran ten trials with a 2 × 2 configuration where the Kilobots activated their motors to move forward without any feedback nor communication with neighbouring modules. This configuration did not allow straight motion and only in one of the trials the robot reached the finish line by first hitting the arena’s wall. In the second experiment, we ran ten trials with a 2 × 2 configuration and a further ten trials with a 3 × 3 configuration, where, this time, the Kilobots ran the same algorithm as used in the experiments with elastic links (hence, communicating with neighbouring modules). In none of the trials, the robot reached the finish line, owing to a combination of poor motion accuracy and speed. A two-tailed Mann–Whitney test with an alpha level of 0.05 revealed that robots with elastic links (2 × 2 and 3 × 3 configurations) outperformed those with rigid links (2 × 2 and 3 × 3 configurations). The trajectories of the rigid links trials are included in Fig. S4 of the Supplementary Information.

### Design considerations

**Fig. 5:**
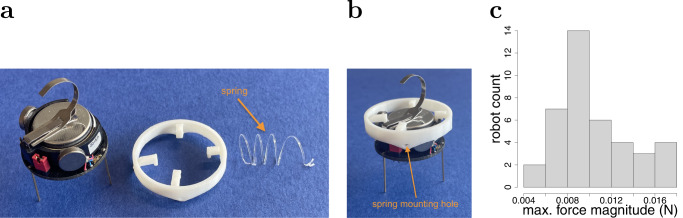
Overview of the module of the Kilobot Soft Robot. **a** Up to four helical springs can be connected to the Kilobot through a 3-D printed circlet. **b** Kilobot equipped with the circlet. The circlet is firmly attached to the Kilobot (i.e. the latter can not rotate within the circlet). Four springs are attached at fixed mounting holes, thereby constraining the movements (and orientation) of the module. **c** Maximum magnitude of force a module can exert onto a single spring (based on 40 robots, each subjected to five independent trials).

The maximum magnitude of force that a single module may exert onto the structure depends on the module’s propulsion capability and ground friction. We experimentally determine this quantity for 40 individual modules (for details, see Section ‘Deformation analysis’ in ‘Methods’). Figure 5c reveals a substantial variation across the modules. Depending on the module used, a single of our springs would extend by about 29% to 90% of its rest length (*L*).

A formal analysis of the elastic links (see Supplementary Note S1 and Section ‘Deformation analysis’ in ‘Methods’) shows that a module in interior position *P*_3_ of the square lattice (i.e. being linked to four springs) could displace not more than 15% to 56% of the aforementioned magnitude (*L*). Moreover, the force profile is found to be non-isotropic (Fig. S5a, b). A module encounters the most resistance if moving directly towards a neighbour, whereas it encounters the least resistance if moving in a direction exactly in between two adjacent neighbours. The former case poses control challenges, as deviations from the desired direction of motion are amplified by the force field, whereas in the latter case the force field has some capability of self-correcting such deviations (see Section ‘Deformation analysis’ in ‘Methods’). This motivates our choice of using a diagonal direction of motion for the square lattice based robots (see Fig. 1b). For the hexagonal lattice based robots (Fig. S2), the module’s orientation (and principle direction of motion) should be chosen as in Fig. S2a.

## Discussion

In this paper, we proposed a novel concept enabling reliable movement to arise in aggregates of highly error-prone modules. The modules are mechanically coupled via deformable links. They assume a lattice based formation, which can be manually reconfigured to create structures of arbitrary scale. To validate the concept in practice, we created a prototype system called Kilobot Soft Robot. Its modules, Kilobots, are linked via springs in a square lattice formation. Each module moves by vibration motors and determines the distance (but not the bearing) of its local neighbours. We presented distributed control algorithms that enable Kilobot Soft Robots to move, shrink, and stretch in size. The algorithms are applicable to both square lattice and hexagonal lattice based robots.

To analyse the performance, we conducted a series of systematic experiments with Kilobot Soft Robots comprising up to 49 modules, organised in a 7 × 7 square lattice. The results show that as the number of modules increased, so did the accuracy of the robot’s motion. The speed of the robot initially decreased though settling to a constant value for robots larger than 4 × 4. Moreover, the Kilobot Soft Robots proved capable of following a given reference trajectory, and of shrinking and stretching their bodies.

Although the modules have highly error-prone and noisy sensing and motion capabilities on their own, these inaccuracies cancel out in large aggregates. Similar averaging phenomena in which many-wrongs outperform best individuals are observed in various domains^5,62–64^, sometimes referred to as the wisdom of crowds. Unlike most previous works on the many-wrongs principle, our work focused on groups of individuals that are mechanically coupled via deformable links. We showed that these links facilitate coherent motion—the Kilobot Soft Robots would no longer move coherently once the mechanical couplings were removed. Notably, the deformable links enable the aggregates to leverage the underlying material properties, and thereby reduce the reliance on (energy-intense) perception, computation, and actuation. These findings could pave the way for realising aggregates of ultra-low-power devices that move and deform in precise ways. Possible applications include robots with stretchable bodies that perform high-resolution monitoring and/or manipulation of surfaces in confined spaces.

This paper presented one of the first realisations of soft-bodied robots composed of large numbers of autonomous and reconfigurable modules. We conducted a rigorous experimental evaluation of a self-propelling modular robot at a large scale, with 49 modules. We examined the question whether soft connections are superior to rigid ones for a modular robot to effectively move across the ground. We tested this hypothesis by conducting control experiments where 2 × 2 and 3 × 3 Kilobot Soft Robots had their elastic links replaced with rigid ones. The rigid robots reached the goal in none of the trials, whereas the elastic robots reached it in 30% and 80% of the trials, respectively.

When connected via rigid links, the modules’ motions are highly constrained, and any disagreements, for example, as a result of sensory noise, may cause the robot to stall. The rigidity of the structure may also impede a module’s ability to contribute effectively to correcting the course of the robot. Note that these observations relate to lightweight ground modules that can exert only limited forces onto each other when compared to the forces required to overcome (static) ground friction. Where (static) frictional forces are negligible (e.g. in vacuum or fluidic environments), efficient collective propulsion could be realised with rigidly-linked modules as well^25,29^. By contrast, when connected via elastic links, the modules are more flexible with respect to their movements, as the constraints are only gradually enforced. Moreover, where the movements cause compression or extension of a spring (relative to rest length), kinetic energy is converted into elastic potential energy and can be released later, helping to restore the original configuration. Ultimately, this process is analogous to relaxation in the presence of an energy potential, which is quadratic due to the nature of the spring forces involved. This relaxation reduces the relative positioning error of the modules, and under ideal conditions reaches a global minimum, ensuring coherence and stability.

We conducted a formal analysis of the forces acting on a module via its elastic links. This revealed that a module encounters maximal resistance while moving from its neutral (resting) position towards a neighbour, and that deviations from such reference direction become amplified by the underlying force field. By contrast, a module encounters minimal resistance while moving towards the “centre” of two adjacent neighbours, and the deviations from such reference direction are reduced by the force field. The findings have important consequences for the design of soft-bodied modular robot aggregates. For square lattice based robots (e.g. Fig. 1), the preferred direction of motion would be along the diagonal, whereas for hexagonal lattice based robots, the preferred direction would be the one indicated in Fig. S2a, as opposed to the one in Fig. S2b.

Future work may consider the presence of external entities that the Kilobot Soft Robot can come in contact with. In particular, it may be possible for the robot to detect the presence, weight, and even the shape of such entities, from the internal deformations alone^65^. Future work could also consider systems that move and deform effectively in 3-D environments, and work towards the miniaturisation of the modules.

## Methods

This section presents the methodology, including details regarding the hardware platform, deformation analysis, algorithms and experimental setup.

### Hardware platform

The modules of the Kilobot Soft Robot are arranged in an *r* × *c* square lattice configuration (see Fig. 1b). The number of rows and columns, *r*, *c*≥1, can be manually reconfigured; they remain constant during a trial. Each module is connected to all other modules in its von Neumann neighbourhood (see Fig. 1b). The links (springs) are elastic.

Each module is based on a Kilobot unit. The module has two vibration motors, allowing it to turn left or right, or move forward. The module is unable to rotate on the spot or move backwards.

The module has an infra-red transceiver pointing towards the bottom, allowing it to exchange messages with other modules in its vicinity in a range of about 15 cm (the corresponding signals bounce off the reflective ground). The spacing between connected modules has been chosen such that a module can communicate with the eight modules in its Moore neighbourhood (see Fig. 1b), even in the presence of moderate distortions. However, due to hardware differences among the modules and communication noise, modules may not always be able to communicate with neighbouring modules, or may receive messages from modules outside their original neighbourhood.

The links between modules are implemented using helical springs. As commercially available springs proved too heavy, custom-made springs were produced from a 500-μm acetate sheet. The sheet was sliced in ~800-μm-thick filaments. The filaments were winded around a tube of 1.4 cm diameter and then heated to assume the helical shape. The resulting springs, shown in Fig. 5a, had a length of ~3.1 cm, a weight of ~0.0425 g, and a spring constant of ~0.6 N/m (measurement average for 20 springs).

To attach the springs to the Kilobots, we designed a circlet (see Fig. 5a, b). The circlet has a diameter of 4 cm and weighs 1.4 g. For comparison, the Kilobot has a diameter of 3.3 cm and weighs 17.2 g. The circlet is mounted on top of the robot. The design offers a firm grasp while avoiding interference with the motors or communication system, and allowing the Kilobot to display its light-emitting diode. The circlet has four small holes equally spaced around the ring. The tangle at the end of the spring allows the user to lace it to each of these four holes. Each module can have a maximum of four springs attached at 45°, 135°, 225°, 315° with respect to the forward motion direction. Once mechanically linked with neighbours, the module’s movements (and orientation) are constrained by the springs which, while providing some flexibility, induce a bias towards a common direction. This constraint is particularly useful because Kilobots do not have any orientation sensor and would otherwise risk drifting towards undesired directions, as reported in ref. ^66^.

### Deformation analysis

This section examines the extent to which the modules’ movement may deform the lattice configuration. It first presents experimental findings regarding the forces that a single module is able to exert with its vibration motors. It then presents theoretical findings regarding the forces that would be needed to deform a local configuration by some specified amount. Finally, it examines the implications of the theoretical findings for the design of robots that shall move coherently.

The maximum magnitude of force that a single module may exert onto the structure depends on the module’s propulsion capability and ground friction. To determine this force, we consider a spring attached with one end to the circlet of a module, which is facing away from the spring, and with the other end to a fixed anchor at the same height. The module is programmed to move forward, thereby extending the spring. We exclusively used modules with a good battery charge (as indicated by a blue or green LED, as opposed to yellow or red). We performed 5 independent trials for 40 different modules, resulting in 200 experimental observations. Figure 5c shows the magnitude of the force (in N) each module was able to exert on the spring (we report the median value of the five trials; deviations for the same module were minimal). Depending on the particular Kilobot used, the force assumed values within 0.0054 N–0.0167 N. These results suggest that there is substantial variation in the actuation capabilities of the individual modules; nevertheless the Kilobot Soft Robot is capable of relatively consistent and accurate propulsion (Fig. 2).

The force that the Kilobot-based modules could produce could allow a single spring to extend by about 0.9 cm to 2.8 cm, corresponding to 29% to 90% of the rest length of the spring (assuming a spring constant of 0.6 N/m and rest length 3.1 cm). However, when part of a square lattice configuration, the movement of any module is constrained by multiple springs.

In the following, we consider a module in interior position *P*_3_, and attached via springs to four stationary neighbours. From our analysis based on first principles (see Note S1 in Supplementary Information), we obtain that by exerting a force of no more than 0.0054 N to 0.0167 N, the module would displace not more than 0.46 cm to 1.73 cm (see Fig. S5a). This compares reasonably well with the length of the springs (~3.1 cm).

Figure S5b shows the force profile generated by the closed-form equation *f*(*ρ*, *θ*) (see Note S1 in Supplementary Information). It reveals a non-isotropic force profile. The focal module, placed in the origin (i.e. with all elastic links being at rest) encounters maximal resistance if moving directly towards a neighbour (due to symmetry, the particular choice of neighbour is not relevant). If moving in a direction exactly in between two adjacent neighbours, however, the module encounters the least resistance. An analysis of the force profile for the hexagonal lattice (see Figs. S2 and S5c) reveals the same—the forces of maximal and minimal magnitude occur when moving towards a neighbour and in between two adjacent neighbours, respectively.

Controlling a module to move towards a direction of maximal resistance is particularly challenging, as deviations become amplified, whereas controlling a module to move towards a direction of minimal resistance is facilitated by the self-corrective nature of the force field. Both of these effects are confirmed by close inspection of the gradients for the square lattice formation in Fig. S5a, where the maxima and minima (in resistance) occur in the horizontal/vertical directions and diagonal directions, respectively. This motivates our choice of using a diagonal direction of motion for the square lattice based robots (see Fig. 1b). For the hexagonal lattice based robots (Fig. S2), the module’s orientation (and principle direction of motion) should be chosen as in Fig. S2a.

### Algorithms

While the individual modules in the Kilobot Soft Robot are mechanically coupled, each of them is still fully autonomous, and acting solely based on local information. This section details the decentralised algorithms that are executed by the modules.

Each module executes an identical set of algorithms. The modules are thus fully inter-changeable. The Kilobot Soft Robot can be arbitrarily reconfigured without reprogramming the modules, due to the modular and scalable design. All algorithms are openly available at https://github.com/ilpincy/argos3-kilobot/blob/softrobot/src/examples/behaviors/KBSR_rebuild.c.

Before deciding if and where to move, a module estimates its location relative to other neighbouring modules. Thereafter, the module determines a motion command. Both algorithms are executed concurrently.

#### Localisation

Each module uses the localisation algorithm to estimate the position of neighbouring modules relative to its own. This task is challenging: although the module can measure the signal strength of an incoming message, and hence estimate the distance of the emitter, it does not know the bearing, that is, the angular position of the emitter in the module’s reference frame. The springs simplify the estimation problem, as the modules of the Kilobot Soft Robot can not be positioned independently. For example, given a Kilobot Soft Robot of more than two modules, it is hardly possible to swap the positions of two modules, while retaining all other modules’ positions.

We assume that at the time of configuring the Kilobot Soft Robot, each module is assigned a unique ID and made aware of the overall robot dimensions (*r* × *c*). The IDs are assigned sequentially throughout the lattice, as indicated in Fig. 1b. Given an ID *i*, and dimensions *r* and *c*, a module can determine the IDs of modules in its neighbourhood. For example, the IDs of modules in the von Neumann neighbourhood are *i* − 1 (for $i\,{{{{{{\mathrm{mod}}}}}}}\,\,c\, \ne \, 1$), *i* + 1 (for $i\,{{{{{{\mathrm{mod}}}}}}}\,\,c\, \ne \, 0$), *i* − *c* (for *i* > *c*), and *i* + *c* (for *i* ≤ *rc* − *c*). Although not considered in our experiments, the Kilobots could autonomously determine the dimensions of the lattice configuration (*r* × *c*), identify their positions within the configuration, and self-assign a globally-unique ID, by running decentralised algorithms such as^58,61^.

Each module has a maximum of eight neighbours (see Fig. 1b). It continuously sends messages via local broadcast to and receives messages from these neighbours at the maximum communication frequency of 2 Hz (messages from modules outside the Moore neighbourhood are discarded). The message is of constant length (nine bytes). It contains the module’s ID (one byte) and its own distance estimates (one byte each) for the eight neighbouring modules (where available). Initially, the module has no estimate of any distance. However, every time a message arrives, the module uses the signal strength to estimate the distance of the emitting module. The module stores all distance estimates in a 9 × 9 matrix, which contains (where available) one entry for every pair of modules in the local neighbourhood. The matrix is reset periodically (every 2 s), thereby removing outdated information (e.g. following a loss of communication). Note that the estimates for pairs (*i*, *j*) and (*j*, *i*) usually differ for *i* ≠ *j*. In the subsequent calculations the average is used where both values are available. Note that the matrix is typically not completely filled, as some neighbours can be out of the communication range of others, or the focal module could be part of the boundary of the lattice structure.

Recall that due to communication (and sensing) being isotropic, a module is unable to determine its orientation (unless when moving relative to its neighbours). However, it can use distance measurements to infer the bearing of one neighbour relative to another, and does so at the beginning of every cycle of the motion control algorithm. Depending on its principle position within the lattice, each module estimates one or two angles (see Fig. 1c), which are used in conjunction with selected distances to determine whether any deformation is present. For example, an interior module (labelled *P*_3_) with ID *i* calculates the angle between neighbours *i* − *c* and *i* − 1, as well as the angle between neighbours *i* + *c* and *i* + 1.

To estimate the angle between two of its neighbours, a module uses the law of cosines. This requires only the pairwise distance estimates (see Fig. S6a in Supplementary Information). In particular, for any triangle with sides *a*, *b*, and *c*, and the angle between sides *a* and *b* denoted by *γ*, the following equation holds: ${d}_{c}^{2}={d}_{a}^{2}+{d}_{b}^{2}-2{d}_{a}{d}_{b}\cos \gamma$, where *d*_*a*_, *d*_*b*_, and *d*_*c*_ denote the distances of sides *a*, *b*, and *c*. Note that ambiguous situations can occur (see Fig. S6b in Supplementary Information). These can be resolved by taking into account the default relative positioning of the modules and their IDs (see Fig. 1c). In fact, due to the presence of physical links, position swaps are unlikely to happen.

#### Motion control

The purpose of the motion control algorithm is to move the Kilobot Soft Robot into a desired direction while maintaining its default shape, the square lattice configuration shown in Fig. 1b.

The Supplementary Information (Algorithm S1) presents the motion control algorithm, which is executed periodically by every module of the robot. The module’s desired direction of motion is denoted by TARGET. It can assume three possible values (forward, left, right), and is interpreted relative to a module’s local reference frame. A specific, possibly dynamically evolving, target direction can be provided. By default, the target direction is forward.

**Fig. 6:**
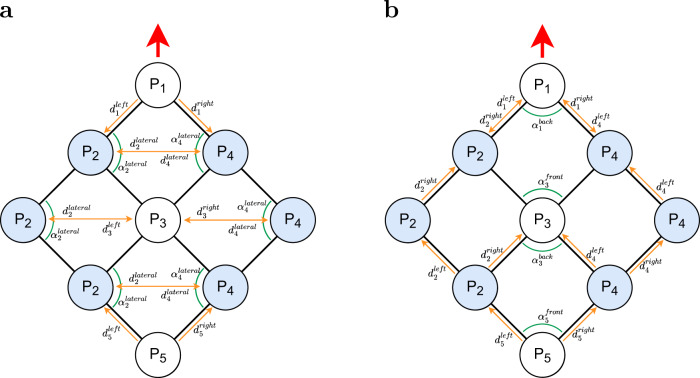
The modules of the Kilobot Soft Robot use their localisation estimates to identify any deviations from the reference shape (square lattice). **a**, **b** show the set of distances and angles considered by Algorithm S1 during the lateral and longitudinal deformation tests, respectively. White and blue colours indicate modules with a symmetric and asymmetric neighbourhood, respectively. The red arrow indicates the Kilobot Soft Robot’s forward direction.

The motion control algorithm uses estimates of the distances and angles shown in Fig. 6. The methods for obtaining these values are described in the ‘Localisation’ section. Distance estimates are updated in parallel. Angle estimates are obtained at the time of use, based on the most recent distance estimates. If, at the time of executing the algorithm, a distance or angle estimate is not available, all expressions that make use of them are evaluated as false. This is particularly relevant for modules at positions *P*_2_ or *P*_4_, as depending on where they reside on the left/right boundary, ${d}_{p}^{{{{{\mathrm{left}}}}}}$ or ${d}_{p}^{{{{{\mathrm{right}}}}}}$ may not be defined (see line 29 of Algorithm S1).

Each module begins the cycle by probing for lateral deformations, and if necessary performs a corrective move (procedure CONTROL-LATERAL-DEFORMATION). The module uses any of its distance and angle variables shown in Fig. 6a. Recall that modules are divided into two groups, depending on whether their neighbourhood is left-right symmetric (*P*_1_, *P*_3_, *P*_5_) or not (*P*_2_, *P*_4_) as illustrated using white and blue discs in Fig. 6. In the former case (lines 8–12 of Algorithm S1), the module tests whether its neighbours on the left and right are about the same distance away, and, where this is not the case, turns towards the neighbour further away. In the latter case (lines 13–21), the module resides in the left or right boundary. The module compares the distance of its (sole) lateral neighbour against a reference value (${d}_{{{{{{{{{\rm{sep}}}}}}}}}_{2}}$), while also evaluating a lateral angle against a reference value of ${\alpha }_{{{{{{{{{\rm{ref}}}}}}}}}_{2}}=9{0}^{\circ }$. For hexagonal lattice configurations, depending on the robot’s default heading with respect to the lattice, ${\alpha }_{{{{{{{{{\rm{ref}}}}}}}}}_{2}}$ would be 120° (see lateral angles in Fig. S2a) or 60° (see lateral angles in Fig. S2b).

Each module then tests whether it had advanced excessively along the longitudinal axis of motion (procedure EXCESSIVE-LONGITUDINAL-ADVANCE of Algorithm S1). To do so, the module uses any of its distance and angle variables shown in Fig. 6b. It compares the distance and angle variables against reference values of ${d}_{{{{{{{{{\rm{sep}}}}}}}}}_{1}}$ and ${\alpha }_{{{{{{{{{\rm{ref}}}}}}}}}_{1}}=9{0}^{\circ }$, respectively. For hexagonal lattice configurations, depending on the robot’s default heading with respect to the lattice, ${\alpha }_{{{{{{{{{\rm{ref}}}}}}}}}_{1}}$ would be 60° (see longitudinal angles in Fig. S2a) or 120° (see longitudinal angles in Fig. S2b).

If a module determines that it has advanced by too far along the longitudinal axis of motion, it cease motion, by turning off its motors for a set duration (line 4 of Algorithm S1). Otherwise, it moves towards the pre-defined direction (forward, left, or right) for a set duration (line 6). To reduce the risk of deadlocks, we recommend choosing conservatively the thresholds that cause modules to cease motion. The inherent noise in the modules’ sensor measurements and the elasticity of the mechanical coupling—enabling neighbours of a stopped member to advance further and eventually pull along the stopped member—contribute as well to the resolution of deadlocks.

Parameter ${d}_{{{{{{{{{\rm{sep}}}}}}}}}_{1}}$ and ${d}_{{{{{{{{{\rm{sep}}}}}}}}}_{2}}$ are ideally chosen as Δ and $\sqrt{2}{{\Delta }}$, where Δ denotes the resting distance between adjacent modules (approx. 7 cm including spring and circlets). For hexagonal configurations, the corresponding values are either both Δ (see Fig. S2a) or Δ and $\sqrt{3}{{\Delta }}$ (see Fig. S2b).

Further parameters are used to control the tolerances (*ϵ*_0_ = 1 cm, *ϵ*_1_ = 0 cm, *ϵ*_2_ = 0.5 cm, *α*_*ϵ*_ = 5°). The length of the time step (*t*_Δ_) is 500 ms.

#### Deformation control

Due to the elasticity of the links between the modules, the Kilobot Soft Robot can modify its shape. This is particularly useful when the robot has to move through complex environments comprising small passages.

By default, the motion control algorithm assumes that the robot’s base structure (a square lattice) is to be maintained. The algorithm can, however, be modified to realise a different reference shape. For example, in the deformation experiments, the robot contracts and then extends again. This can be achieved by changing the reference angles, ${\alpha }_{{{{{{{{{\rm{ref}}}}}}}}}_{1}}$ and ${\alpha }_{{{{{{{{{\rm{ref}}}}}}}}}_{2}}\!$, which are by default (90°), using an offset *α*. Consider the situation depicted in Fig. 6. The modules represented using white discs would use ${\alpha }_{{{{{{{{{\rm{ref}}}}}}}}}_{1}}-\alpha$ as the reference angle, whereas the modules represented using blue disk would use ${\alpha }_{{{{{{{{{\rm{ref}}}}}}}}}_{2}}+\alpha$ as the reference angle. For the deformation experiments, we chose *α* = 50°.

### Experimental setup

The experiments are conducted on a flat, bounded 200 cm × 200 cm arena made of whiteboard Perspex^ⓒ^ Frost matt acrylic material (Moonlight White S2 1T41). In two of the experiments, feedback related to the position with respect to a reference trajectory is provided to one of the robot’s modules. This is realised using the Augmented Reality for Kilobots (ARK) technology^67^. ARK makes it possible to have virtual sensors on Kilobots. It comprises an array of overhead cameras for real-time position tracking, a base control station, and an array of overhead infra-red transmitters to send in real-time addressed messages to each augmented Kilobot. ARK is openly available and has been proven to support swarms comprising hundreds of robots^68^. In all experiments, ARK is used to record the positions of the modules for post-analysis.

At the beginning of each trial, the modules of the Kilobot Soft Robot were positioned such that the connecting springs were at rest. This resulted in a centre-to-centre distance between connected Kilobots of ~7 cm. The experiments were conducted with fully charged Kilobots. The Kilobot’s vibration motors were calibrated for each set of experiments.

Video recordings from the experimental trials are available at 10.15131/shef.data.22269319.

## Supplementary information

Supplementary Information

Description of Additional Supplementary Files

Movie S1

## Data Availability

Video recordings of the experiments are available at: 10.15131/shef.data.22269319. Data for the force profile plots (Fig. S5a–c) are available via the same link.

## Code availability

The Kilobot Soft Robot algorithms are openly available at https://github.com/ilpincy/argos3-kilobot/tree/softrobot/ and https://github.com/ilpincy/argos3-kilobot/blob/softrobot/src/examples/behaviors/KBSR_rebuild.c. The computer programs used to calculate the data for the force profile plots (Fig. S5a–c) are available on 10.15131/shef.data.22269319.
