# Supplementary material for: Coherent movement of error-prone individuals through mechanical coupling
Source: Nat Commun. 2023 Jul 18;14:4063. doi: 10.1038/s41467-023-39660-6 (PMC10354013; doi:10.1038/s41467-023-39660-6)
Supplement: Supplementary file 1 — Supplementary Information [file 41467_2023_39660_MOESM1_ESM.pdf]

Supplementary Information for

Coherent movement of error-prone individuals through mechanical coupling

Federico Pratissoli,[1,2] Andreagiovanni Reina,[2,3] Yuri Kaszubowski Lopes,[4]
Carlo Pinciroli,[5] Genki Miyauchi,[2] Lorenzo Sabattini,[1] Roderich Groß[2]

[1]Department of Sciences and Methods for Engineering, University of Modena and Reggio Emilia, Italy

[2]Department of Automatic Control and Systems Engineering, The University of Sheffield, UK

[3]IRIDIA, Université Libre de Bruxelles, Belgium

[4]Department of Computer Science, Santa Catarina State University, Brazil

[5]Department of Robotics Engineering, Worcester Polytechnic Institute, MA, USA

This PDF file includes:

Table S1. Percentage of successful trials in the straight motion experiment.

Figure S1. Graphically enhanced version of Figure 1a, showing side-view of the 7×7 Kilobot Soft Robot.

Figure S2. Illustration of the Kilobot Soft Robot in hexagonal lattice configurations.

Figure S3. Trajectories obtained in physical experiment in which the elastic links of 2×2 Kilobot Soft Robots are removed.

Figure S4. Physical experiment in which the elastic links of Kilobot Soft Robots are replaced by rigid links.

Figure S5. Findings from theoretical force analysis of a focal module attached to $n = 4, 6$ stationary neighbours.

Figure S6. Illustration of the method to determine the bearing of one neighbour relative to another.

Method S1. Motion control algorithm.

Note S1. A study of the force profile experienced by a robot linked to n stationary static neighbours.

Other supplementary information for this manuscript includes the following:

Movie S1 (.mp4 format). Excerpts from physical robots trials.

Supplementary Tables

Table S1: Percentage of successful trials in the experiments where a Kilobot Soft Robot, of size S, is tasked to move forward by $120\,\text{cm}$ in the absence of any external feedback. 10 trials per robot size.

Robot size, S	Success rate (in percent)
1×1	40
2×2	30
3×3	80
4×4	90
5×5	90
6×6	90
7×7	90

Supplementary Figures

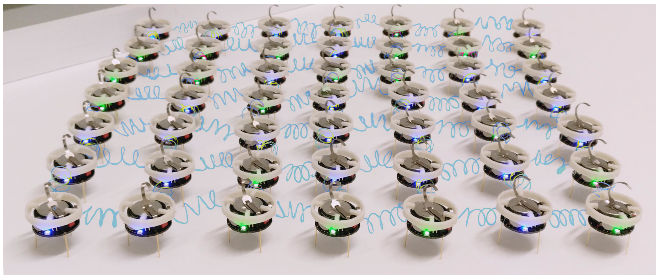

Figure S1: Graphically enhanced version of Figure 1a where the transparent springs are superimposed with blue colour. Shown is a side-view of the 7×7 Kilobot Soft Robot.

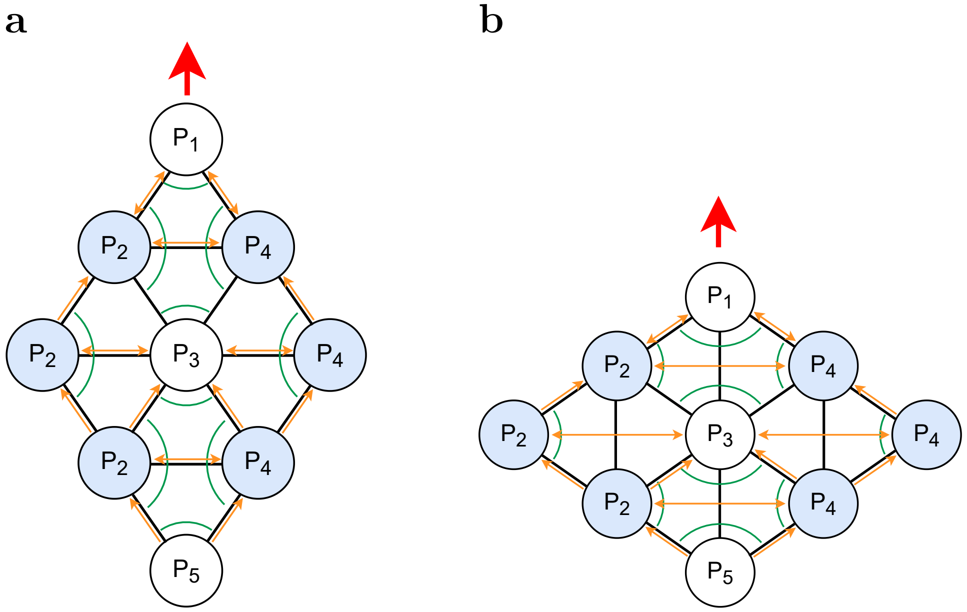

Figure S2: The algorithms are applicable to configurations where the Kilobot modules are arranged in a hexagonal lattice. **a** and **b** show two such configurations, which differ in the robot's principle direction of motion (i.e. relative to the orientation of the hexagonal lattice), which is indicated by a red arrow. The motion control algorithm is identical to the one for square lattice robots, expect for references values α_{ref_1}, α_{ref_2}, d_{sep_1}, and d_{sep_2}, against which the angle and distance variables are compared (for more details, see the Motion control section in the Methods).

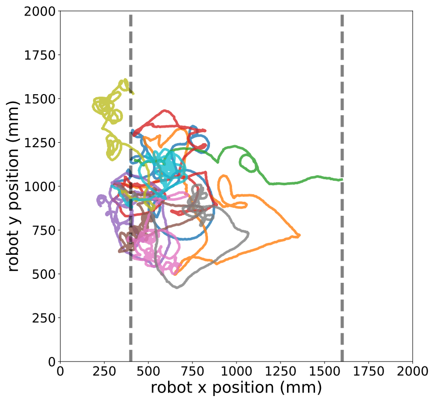

Figure S3: Trajectories obtained in physical experiment in which the elastic links of 2×2 Kilobot Soft Robots are removed (10 trials). The robots are tasked to move straight for 120 cm in the absence of external feedback. As executing the default algorithm, each module still responds to the feedback provided by other modules.

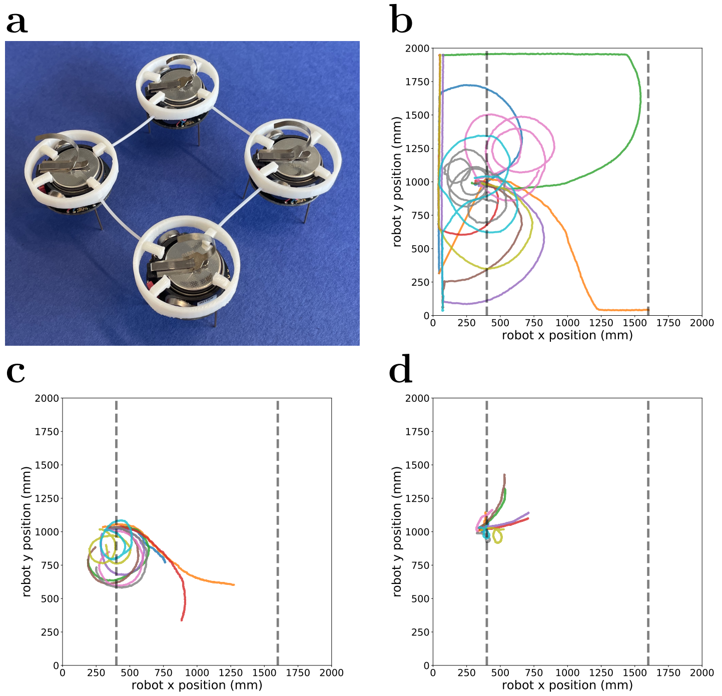

Figure S4: Physical experiment in which the elastic links of Kilobot Soft Robots are replaced by rigid links. The task is to move straight for 120 cm in the absence of external feedback. **a** A picture of a 2×2 Kilobot Soft Robot with rigid links. **b** Trajectories obtained with 2×2 configuration where Kilobots move straight without communicating with other robots. (**c**-**d**) Trajectories obtained with **c** 2×2 and **d** 3×3 configurations where Kilobots run the same algorithm as used in the experiments with the elastic links (hence, communicating with neighbouring modules and adapting the motion behaviour accordingly).

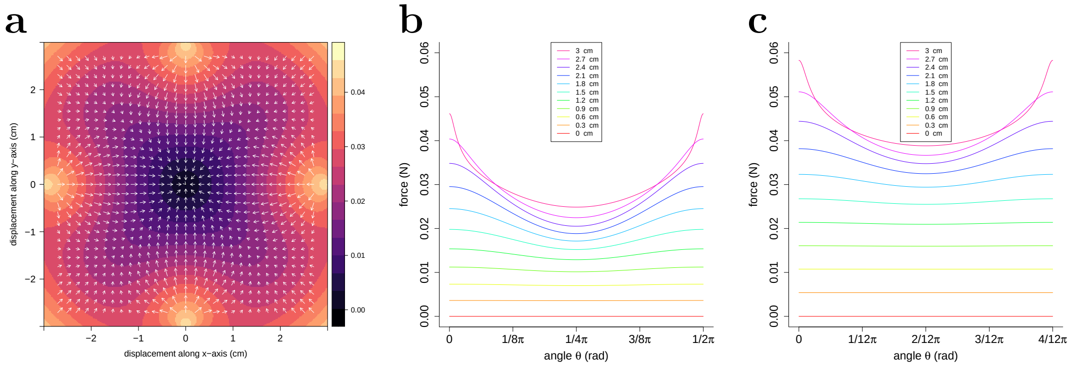

Figure S5: Results from theoretical force analysis of a focal module that is elastically coupled with n uniformly distributed, stationary modules (see Note S1 of Supplementary Information). **a** Magnitude of force, and gradient thereof, based on the focal's module displacement relative to its rest position in Cartesian coordinates (assuming a square lattice configuration, $n = 4$). **b** Force profile based on the focal's module displacement relative to its rest position in polar coordinates (generated by closed-form equation, $f(\rho, \theta)$, assuming a square lattice configuration, $n = 4$). The x axis is θ and the y axis is the force magnitude due to different values of ρ. Due to symmetry, we only consider values of θ from 0 (first neighbour) to $\pi/2$ (second neighbour). **c** Force profile as in (a), however, assuming a hexagonal lattice configuration, $n = 6$. The first and second neighbours are at $\theta = 0$ and $1/3\pi$, respectively.

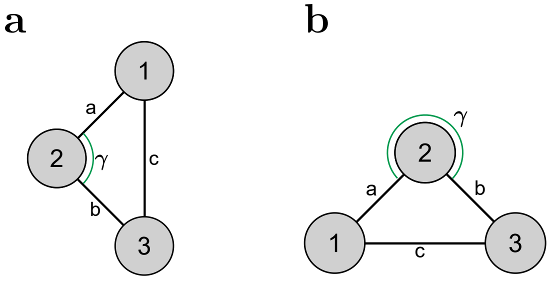

Figure S6: The module's neighbourhood analysis. **a** Module 2 can estimate the bearing of neighbour 1 relative to neighbour 3 (γ). It does so using the law of cosines, which relates the angle to the lengths of sides a, b, and c of the triangle (for details, see text). However, module 1 could as well be in the position shown in **b**. The localisation ambiguity can be resolved by taking into account the position of modules in the undistorted lattice, where all angles are right angles. Note that the magnitude of angular distortions is limited by the mechanical coupling among the modules. Therefore, module 1 is not likely to reside in the position shown in **b**.

Supplementary Methods

Algorithm S1 Motion control algorithm for a module at position $P_p, p \in \{1, 2, \ldots, 5\}$ as specified in Figure 1c. Distances and angles are those shown in Figures 6 (for square lattice configurations) and S2 (for hexagonal lattice configurations), respectively.

.

procedure MOTION
  CONTROL-LATERAL-DEFORMATION()
  if EXCESSIVE-LONGITUDINAL-ADVANCE() **then**
    stop movement for time t_Δ
  else
    move in `TARGET` direction for time t_Δ
procedure CONTROL-LATERAL-DEFORMATION
  if $p \in \{1, 3, 5\}$ **then** ▷ head (P_1), interior (P_3), or tail (P_5)
    if $d_p^{\text{right}} > d_p^{\text{left}} + \epsilon_0$ **then**
      turn right for time t_Δ
    else if $d_p^{\text{left}} > d_p^{\text{right}} + \epsilon_0$ **then**
      turn left for time t_Δ
  else
    if $p = 2$ **then** ▷ left boundary (P_2)
      `ROBOT_CENTRE` = right
    else ▷ right boundary (P_4)
      `ROBOT_CENTRE` = left
    if $(\alpha_p^{\text{lateral}} < \alpha_{\text{ref}_2} - \alpha_\epsilon) \vee (d_p^{\text{lateral}} > d_{\text{sep}_2} + \epsilon_2)$ **then**
      turn towards `ROBOT_CENTRE` for time t_Δ
    else if $(\alpha_p^{\text{lateral}} > \alpha_{\text{ref}_2} + \alpha_\epsilon) \vee (d_p^{\text{lateral}} < d_{\text{sep}_2} - \epsilon_2)$ **then**
      turn away from `ROBOT_CENTRE` for time t_Δ
procedure EXCESSIVE-LONGITUDINAL-ADVANCE
  if p=1 **then** ▷ head (P_1)
    return $(\alpha_p^{\text{back}} < \alpha_{\text{ref}_1} - \alpha_\epsilon) \wedge (\min(d_p^{\text{left}}, d_p^{\text{right}}) > d_{\text{sep}_1} + \epsilon_1)$
  if p=3 **then** ▷ interior (P_3)
    return $(\alpha_p^{\text{front}} > \alpha_{\text{ref}_1} + \alpha_\epsilon) \vee (\alpha_p^{\text{back}} < \alpha_{\text{ref}_1} - \alpha_\epsilon)$
  if p=5 **then** ▷ tail (P_5)
    return $(\alpha_p^{\text{front}} > \alpha_{\text{ref}_1} + \alpha_\epsilon) \wedge (\max(d_p^{\text{left}}, d_p^{\text{right}}) < d_{\text{sep}_1} - \epsilon_1)$
  return $(d_p^{\text{right}} < d_{\text{sep}_1} - \epsilon_1) \vee (d_p^{\text{left}} < d_{\text{sep}_1} - \epsilon_1)$ ▷ left/right boundary (P_2, P_4)

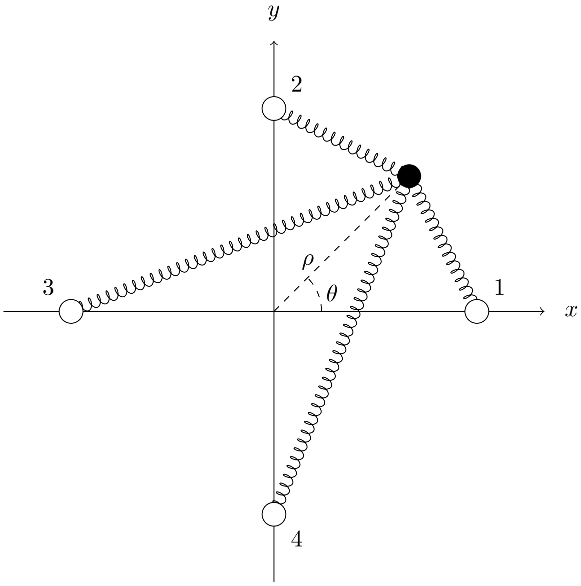

Figure S7: A diagrammatic depiction of the linkage configuration considered in Note S1.

Supplementary Notes

Note S1: Elastic Linkage Force Profile

We report a closed-form solution of the force profile of elastic linkage assuming a square lattice formation. We consider a module connected to $n > 1$ neighbours. Without loss of generality, the neighbours are symmetrically arranged at fixed distances L from the origin, where L denotes the rest length of the springs. Figure S7 depicts the situation for $n = 4$.

The focal robot's position can be expressed as $\mathbf{x} = \rho \begin{bmatrix} \cos\theta \\ \sin\theta \end{bmatrix} = \begin{bmatrix} \rho\cos\theta \\ \rho\sin\theta \end{bmatrix}$, where ρ is the distance from the origin (the rest position with zero net force) and θ is the angle with respect to the x axis. Let $\mathbf{y_i}$ denote the position of neighbour i, that is $\mathbf{y_i} = L \begin{bmatrix} \cos(\frac{i}{n}2\pi) \\ \sin(\frac{i}{n}2\pi) \end{bmatrix} = \begin{bmatrix} L\cos(\frac{i}{n}2\pi) \\ L\sin(\frac{i}{n}2\pi) \end{bmatrix}$, for $i = 0, 1, \ldots, n-1$.

The force profile we seek is a function, $\mathbf{f}(\rho, \theta)$, that expresses the force required to retain the focal module at $\begin{bmatrix} \rho\cos\theta \\ \rho\sin\theta \end{bmatrix}$, where frictional forces are assumed negligible. It represents the force by which the module would have to propel to remain stationary, and is opposite the sum

of force contributions from the linkages to its neighbours.

Let $\mathbf{d_i} = \mathbf{x} - \mathbf{y_i} = \begin{bmatrix} \rho\cos\theta - L\cos\left(\frac{i}{n}2\pi\right) \\ \rho\sin\theta - L\sin\left(\frac{i}{n}2\pi\right) \end{bmatrix}$.

Using the law of cosines, we obtain

$$|\mathbf{d_i}| = \sqrt{\rho^2 + L^2 - 2\rho L\cos\left(\frac{i}{n}2\pi - \theta\right)}$$

Let $h_i = \frac{|\mathbf{d_i}| - L}{|\mathbf{d_i}|}$. We assume $\mathbf{x} \neq \mathbf{d}_i$, hence, these fractions are well defined.

Using Hooke's law, the force acting on the focal module can be obtained as

$$\begin{aligned} \mathbf{f}(\rho,\theta) &= k\sum_i h_i \mathbf{d_i} \\ &= k\sum_i h_i \begin{bmatrix} \rho\cos\theta - L\cos\left(\frac{i}{n}2\pi\right) \\ \rho\sin\theta - L\sin\left(\frac{i}{n}2\pi\right) \end{bmatrix} \end{aligned}$$

where k is the spring constant. It follows that

$$f_x(\rho,\theta) = k\sum_i h_i\left[\rho\cos\theta - L\cos\left(\frac{i}{n}2\pi\right)\right] \quad (1)$$

$$= k\left[h\rho\cos\theta - L\sum_i h_i\cos\left(\frac{i}{n}2\pi\right)\right] \quad (2)$$

and

$$f_y(\rho,\theta) = k\sum_i h_i\left[\rho\sin\theta - L\sin\left(\frac{i}{n}2\pi\right)\right] \quad (3)$$

$$= k\left[h\rho\sin\theta - L\sum_i h_i\sin\left(\frac{i}{n}2\pi\right)\right], \quad (4)$$

where $h = \sum_i h_i$.

The magnitude of the force, $f(\rho,\theta) = |\mathbf{f}(\rho,\theta)|$, is given by

$$f(\rho,\theta) = \sqrt{f_x^2(\rho,\theta) + f_y^2(\rho,\theta)} \quad (5)$$

For $n = 4$, we obtain

$$
\begin{aligned}
f &= \sqrt{f_x^2(\rho,\theta) + f_y^2(\rho,\theta)} \\
&= \sqrt{k^2\left[h\rho\cos\theta + L(h_2 - h_0)\right]^2 + k^2\left[h\rho\sin\theta + L(h_3 - h_1)\right]^2} \\
&= k\sqrt{h^2\rho^2 + L^2(h_2 - h_0)^2 + L^2(h_2 - h_0)^2 + L^2(h_3 - h_1)^2 + 2L(h_2 - h_0)h\rho\cos\theta + 2L(h_3 - h_1)h\rho\sin\theta} \\
&= k\sqrt{h^2\rho^2 + L(h_2 - h_0)\left[L(h_2 - h_0) + 2h\rho\cos\theta\right] + L(h_3 - h_1)\left[L(h_3 - h_1) + 2h\rho\sin\theta\right]}
\end{aligned}
$$

A plot of the force profile for $n = 4$ is reported in Fig. S5b. The plot shows that, for small distances ρ, the force profile is approximately isotropic. As the module moves further away from the origin, the force profile becomes highly non-linear with force maxima corresponding to the locations of the four neighbours. In other words, a module encounters the largest resistance when moving from the origin towards any of its neighbours. This force profile is confirmed by the magnitude heat map plot shown in Fig. S5a.
