# Supplementary material for: Coherent movement of error-prone individuals through mechanical coupling
Source: Nat Commun. 2023 Jul 18;14:4063. doi: 10.1038/s41467-023-39660-6 (PMC10354013; doi:10.1038/s41467-023-39660-6)
Supplement: Supplementary file 2 — Description of Additional Supplementary Files [file 41467_2023_39660_MOESM2_ESM.pdf]

Description of Additional Supplementary Files

Movie S1: (.mp4 format). Excerpts from physical robots trials.
